# Exploring the Pharmaceutical Care of Pharmacists in China During COVID-19—A National Multicenter Qualitative Study

**DOI:** 10.3389/fpubh.2021.797070

**Published:** 2022-01-27

**Authors:** Mingxia Wang, Zhao Yin, Wan Zhang, Xuedong Jia, Shuzhang Du, Jun Li, Xiaojian Zhang

**Affiliations:** Department of Pharmacy, The First Affiliated Hospital of Zhengzhou University, Zhengzhou, China

**Keywords:** COVID-19, China, pharmacists, pharmaceutical care, qualitative study

## Abstract

**Background:**

Pharmacists are one of the coronavirus disease-2019 (COVID-19) treatment team members in China, yet only a few single-center studies have described the work experience of pharmacists during the pandemic.

**Purpose:**

This study aimed to explore in-depth experiences of hospital pharmacists providing pharmacy services during the COVID-19 pandemic in China on a national scale.

**Methods:**

This is a nationwide multicenter qualitative study that used the purposive sampling method. Semi-structured interviews were conducted with 11 pharmacists from large-scale tertiary hospitals in representative provinces of mainland China. The Colaizzi seven-step method was applied to analyze the interview data.

**Results:**

Eleven semi-structured interviews were conducted. Each interview lasted 25–70 min. By analyzing the work experiences of pharmacists in COVID-19 designated treatment hospitals, five descriptive themes were categorized: (1) drug supply service; (2) routine clinical pharmacy services; (3) expanded pharmacy services during the epidemic; (4) drug management loopholes; (5) areas of improvements of pharmacy services during a pandemic.

**Conclusion:**

During the COVID-19 epidemic, Chinese hospital pharmacists played various vital roles. However, there were loopholes in managing narcotic drugs, psychotropic drugs, and donated drugs. The study uncovered areas of improvement in pharmacy services during the pandemic. The emergency response capacity of hospital pharmacists should be continuously improved in the future.

## Strengths and Limitations of this Study

- This study is the first nationwide qualitative studies to describe the implementation of pharmacy services by hospital pharmacists in China during the COVID-19 pandemic.
- The present study provides an in-depth description of the practical experiences of pharmacists who worked at COVID-19 designated treatment hospitals.
- The study has limitations owing to qualitative research design. The participant number was small, leading to a potential risk of selection bias.

## Introduction

Since December 2019, the cumulative number of reported cases of coronavirus disease 2019 (COVID-19) worldwide has now exceeded 230 million, with more than four million deaths (as of October 12, 2021) (1). COVID-19 infections have triggered a global public health crisis that has lasted nearly 2 years (2, 3). After the Chinese government initiated a nationwide emergency response to the COVID-19 outbreak, hospital pharmacists quickly went to the front line to join physicians and nurses in fighting against the pandemic (4, 5). Compared to the clinical pharmacy in the USA, which has been developing for almost 60 years, clinical pharmacy began in China only in 2005. Despite this, rapid development in clinical pharmacy has been achieved under the support of China's Ministry of Health. Pharmacists in China are starting to provide more patient-centered pharmaceutical care activities instead of focusing solely on drug distribution and dispensing. The role of hospital pharmacists progressed during the pandemic in China. Hospital pharmacists focused on drug supply assurance at the early stage of the outbreak. After a brief panic period, they gradually provided clinical pharmacy services such as drug consultation. As the pandemic progressed, hospital pharmacists expanded traditional clinical pharmacy services into emerging areas of pharmacy services, for example, internet pharmacy services (6, 7).

The International Pharmaceutical Federation (FIP) published guidelines on adapting and expanding pharmacy services during the COVID-19 pandemic (8). The ongoing pandemic has disrupted the health care system, creating challenges for health care workers and patients alike. Ambulatory care pharmacists helped fill gaps in access to primary care services by practicing at the top of their skillset through face-to-face and telehealth services during the pandemic (9). A multinational European qualitative study described a range of service adaptations and adoption of novel pharmacists' roles to prevent and mitigate the public health impact of the pandemic (10). In Australia, pharmacists provided funded drug review services via telemedicine to ensure that vulnerable patients still had access to this vital service (11). Pharmacy professionals have played an integral role in the fight against the COVID-19 pandemic, and these pharmaceutical care activities have relieved pressure on the health system (12–14).

Our single-center qualitative study (15) showed that Chinese pharmacists participated in various emergency response efforts during the COVID-19 outbreak. These efforts included supplying emergency drugs, providing pharmaceutical care, and disseminating knowledge of COVID-19 and potential drug treatments. In the study, we noted that compared with developed countries, the emergency response capacity of Chinese pharmacists to respond to public health crises is still inadequate in the following aspects: (1) at the government level, there is a lack of a national pharmacy emergency plan for public health crises; (2) at the pharmacy department level, there are imperfect emergency response mechanisms and inadequate drug distribution mechanisms; and (3) the clinical service level of pharmacists is lacking compared with that of other front-line clinical staff. However, there is a deficit of studies to date examining core services provided by clinical pharmacists in COVID-designated hospitals in China during the pandemic. This study aimed to explore the views and experiences of clinical pharmacists in China regarding their provision of clinical pharmacy services during COVID-19, focusing on learning points for future public health crises.

## Methods

### Design

This study consisted of semi-structured qualitative interviews with pharmacists working at COVID-designated hospitals in China. The study is reported following the Consolidated Criteria for Reporting Qualitative Studies (COREQ) checklist.

### Sampling and Recruitment

The purposive sampling method was used to recruit participants. To meet inclusion criteria, individuals had to be hospital pharmacists or pharmacy directors who provided clinical or administrative services at designated COVID-19 treatment hospitals and were willing to share their views and work experiences. Designated COVID-19 hospitals across geographical locations at the initial COVID outbreak in China were targeted for participants' recruitment. These hospitals are all tertiary care medical centers with advanced clinical pharmacy practice. The hospitals are located in the less developed regions (central and west China), developed regions (east and south China), and traditional industrial regions in the northeast of China. Individuals interested in participating in this unpaid study were asked to sign a consent form (If an interview was conducted over the cellphone, the written consent form will be obtained by mail). The research team then contacted those agreeing to participate to schedule an interview. Sampling and recruitment continued until achieving data saturation.

### Ethical Considerations

The Institutional Review Board of the First Affiliated Hospital of Zhengzhou University approved the study protocol (2020-KY-224). Participants' rights to informed consent and privacy were protected.

### Data Collection and Analysis

Interviews (one-on-one telephone or face-to-face) were conducted in Chinese by members of the research team consisting of clinical pharmacists and pharmacy directors with qualitative research experience. All interviews took place over May-November 2020. The average duration of the interviews was 35 min, ranging from 25 to 70 min. With participants' consent, each interview was digitally recorded and transcribed. Any identifiers were removed, and an anonymous code was assigned to each participant.

Topic guide questions were developed based on published studies of pharmacy services during the pandemic and our previous published single-center study (15). Topic guide questions had three primary open-ended questions: (1) What tasks did pharmacists undertake in fighting the COVID-19 outbreak? (2) What were the differences between your work content and style during the outbreak compared with regular times? (3) What could hospital pharmacists do differently to improve services if time could turn back? Follow-up questions were asked based on the responses provided by the individual during the interview. The topic guide (Supplementary Box 1) was refined through discussions amongst the research team after pilot interviews with three participants.

The analytical process commenced by listening to interview recordings and repeated readings of the transcripts during the transcribing process. Audio recordings were transcribed verbatim in Chinese by the research team within 24 h and reviewed by two researchers to ensure accuracy. The transcriptions were translated into English and back-translated into Chinese to ensure consistency. Two team members independently conducted data analysis to accurately interpret the recordings and provide an in-depth description of the data. The Colaizzi seven-step (16) method was used for data analysis ((1) Transcribing all recorded data; (2) Extracting significant descriptions; (3) Creating initial meanings; (4) Refining themes; (5) Describing each theme in detail; (6) Identifying the fundamental structure; (7) Returning analyzed data back to participants). NVivo software was used to analyze the raw data. The raw data were encoded and formulated into themes and subthemes. Representative statements from interviewees were selected.

### Trustworthy

Three specific measures were adopted to ensure the credibility of the study. Investigators maintained close contact with the supervising expert who addressed issues and challenges during the study, communicated with the interviewees after data transcribing to confirm that the investigators' understanding matched what the interviewees wanted to convey, and followed the operational procedures and requirements of the qualitative research methodology.

## Results

Fifteen respondents were invited from 15 hospitals, and 11 completed the interviews (four declined or were interrupted during the interviews). Most of the participants (*n* = 8) were male, and the mean age was 45 years old (32–55). All had a bachelor's degree or higher in pharmacy. Hospital practice experience (i.e., years qualified as a pharmacist) averaged 21.8 years (9–35 years). Among the participants, there was the first team of pharmacists who worked at a mobile cabin hospital and the first batch of pharmacy directors who coordinated pharmacy services at the epicenter of the outbreak. The baseline characteristics of the participants are shown in Table 1.

**Table 1:** Socio-demographic characteristics of study participants.

**Variables**	**Frequency (N)**
**Gender**	
Male	8
Female	3
**Age (in years)**	
31–40	4
41–50	1
>50	6
**Positions**	
Director	7
Deputy director	0
Group leader	2
Pharmacist/clinical pharmacist	2
**Education level**	
Bachelor	2
Master and above	9
**Years of practice**	
<10	3
11–20	2
21–30	1
>30	5
**Hospital grade**	
Grade IIIA^*^	11

* *The highest level of hospitals in China*.

After data aggregation, five descriptive themes were identified: (1) drug supply service; (2) routine clinical pharmacy services; (3) expanded pharmacy services during the epidemic; (4) drug management loopholes; (5) areas of improvements of pharmacy services during a pandemic.

### Theme 1: Drug Supply Service

All pharmacists (*n* = 11) mentioned the importance of drug supply (conventional and emergency drugs) during the epidemic, particularly the supply of emergency drugs. During the pandemic, production or transportation disruptions led to drug shortages. Participants described measures taken to ensure the drug supply at each participating hospital.

#### Subtheme 1.1-Establish Emergency Pharmacy or Isolated Ward Pharmacy

In the early stage of the outbreak, several makeshift hospitals (cabin hospitals) were established in the epicenter, Wuhan, China. Temporary pharmacy rooms were quickly set up by local pharmacists or pharmacists outside of Wuhan who came to support them. In addition, pharmacists also set up isolation ward pharmacies to minimize transmissions in COVID-19 designated hospitals throughout China.

“*It was required that drugs must be in place within 4 h when emergency pharmacies were set up in the makeshift hospital. There were only 4 h for us to prepare everything from the time we received the notice”. (P4)*

“*As professional pharmacists, we knew how to get medicines well-preserved, classified, stored, and used, especially medicines with special requirements. Pharmacists played critical roles in managing medications in makeshift hospitals”. (P1)*

“*We established a medicine cabinet for each isolation ward in *<*3 days. Resuscitation drugs that might be used and commonly used drugs were supplied according to the weekly projected usage”. (P2)*

#### Subtheme 1.2-Develop and Update a Catalog of Emergency Drugs

At the initial stage of the outbreak, creating an emergency drug supply list was challenging as too many unknowns about COVID-19. Pharmacists utilized their professional expertise to develop and update their hospitals' emergency drug supply lists.

“*We provided a catalog of around 170 essential drugs at the beginning which increased to nearly 350 later on.” (P7)*

“*We pharmacists were involved in the development of the COVID drug formulary in which we proposed hundreds of amendments. Hospitals all followed this formulary at first; only later did the national program come out.” (P4)*

“*The pharmacists had to draw up a catalog of medicines for routine use and compared it with the doctors' catalog.” (P5)*

#### Subtheme 1.3-Emergency Drug Dispatch and Regular Drug Supply

By the state's requirements, pharmacists' primary job during the epidemic was to supply drugs from the emergency protection catalog to the medical teams rushing to the epicenter, Wuhan.

“*During that time, our pharmacy staff was on standby, medicines were immediately prepared at short notice, at night and during the day, and even our pharmacist worked into the early morning hours one day to get all the medicines ready for the emergency medical teams.” (P1)*

“*The management of suppliers should be strengthened in general, and suppliers should be assessed and managed. The drug distribution suppliers that worked with the pharmacy department were very qualified and competent.” (P3)*

#### Subtheme 1.4-Conducting In-hospital Formulation Production

Some hospitals in China are qualified to produce in-hospital medicinal preparations. Many hospital pharmacists made traditional Chinese medicinal preparations (TCMP) during the epidemic. Some also conducted research and development work on new TCPMs or western medication formulations believed in providing prevention and treatment for COVID at the outbreak.

“*We were making two preparations specifically for COVID-19, which we called Antiviral I and II for short, with the professional name being Yellow Flower Ankara.” (P4)*

“*Hydroxychloroquine was made into a spray, and we obtained a provincial research project” (P5)*

“*The TCMPs were called herbs I, II, and III, which were particularly effective and helped boost the immune system, as well as aiding digestion.” (P9)*

### Theme 2: Routine Clinical Pharmacy Services

On top of ensuring drug supply, hospital pharmacists also provided routine clinical pharmacy services during multidisciplinary team consultations and initiated antimicrobial stewardship.

#### Subtheme 2.1-Join the Multidisciplinary Team

As multidisciplinary team (MDT) member, pharmacists used their pharmacy knowledge to develop individualized medication regimens.

“*Some advice related to therapeutic drug monitoring for priority patients had played a role.” (P4)*

“*A previous patient developed transient deafness, and her doctor would ask the pharmacist if there was any possibility that adverse drug reactions or interactions could have caused it.” (P8)*

#### Subtheme 2.2-Provide Medication Advice

Medication counseling services were the most highly engaged clinical pharmacy service mentioned by the pharmacists interviewed. These included drug contraindications, interactions, and adverse reaction management.

“*After the drug catalog was completed, clinical pharmacists conducted data analysis (including drug compatibility, drug interactions, and contraindications) of the drugs in our catalog in a very short time. Twelve recommendations were finally developed.” (P5)*

“*Pharmacists had created two sets of tables, one on the drug compatibilities and one on the possible interactions of drugs.” (P8)*

“*I found two adverse reactions in the fever ward and adjusted the medication after talking to the doctor. I found this very rewarding as well.” (P2)*

#### Subtheme 2.3-Initiating Antimicrobial Stewardship

In China, clinical pharmacists have relatively solid antimicrobial knowledge. In our study, pharmacists repeatedly mentioned using their antimicrobial knowledge to participate in multidisciplinary consultations or providing antimicrobial stewardship to patients.

“*At that time, the COVID-19 might not be clearly understood. Physicians used more antimicrobial drugs. After pharmacists entered the isolation ward, they promoted the rational use of antibacterial drugs.” (P1)*

### Theme 3: Expanded Pharmacy Services During the COVID-19 Epidemic

After the first phase of panic, pharmacists began to think about how to expand their pharmacy services in response to the changing situation of the epidemic. In this regard, hospital pharmacists primarily engaged in managing drug therapies, disseminating disease states and medication knowledge for COVID-19 patients, providing pharmacy support for medical teams, and starting outpatient drug delivery services through the internet.

#### Subtheme 3.1-Participate in the Discussion and Formulation of Medication Regimens for Hospitalized Patients With COVID-19

Some clinical pharmacists described their deep involvement in managing COVID patients, such as attending MDT rounds, face-to-face communication with patients, and participating in many aspects of therapeutic drug management.

“*Pharmacists went to the ward to check the medical records and attend consultations regularly. Clinical pharmacists specializing in anti-infection also went with the treatment group from the China-Japan Friendship Hospital to answer questions about medications.” (P4)*

”*After entering the isolation ward, I chatted with each COVID-19 patient for about 5*–*10 min, which could increase the confidence of the patients. Also, as a pharmacist, I paid attention to the drip rate of the infusion and whether the medicine should be light protected.” (P2)*

#### Subtheme 3.2-Using New Media to Promote Science

During the epidemic, pharmacists carried out science promotion for the public through new social media such as WeChat public numbers, TikTok, and Weibo. The content of the science promotion primarily included guidance on the use of medicines for common and chronic diseases, introduction to the use of common drugs, and knowledge on the prevention and control of the COVID-19 epidemic.

“*There were a lot of public issues… We wrote almost 50 popular science articles on COVID-19.” (P5)*

“*There was also a column on TikTok for pharmacists, which would be targeted to push out some instructional videos.” (P6)*

#### Subtheme 3.3-Pharmacy Support for Medical Teams Working at the Epicenter

Pharmacists provided pharmacy support to medical teams working at the epicenter, Wuhan, through WeChat or cellphones. Participants stated that they conducted nationwide pharmacy discussions by establishing online groups to answer questions raised by medical teams.

“*The various medical teams found some medication problems on the front line every day; we talked with them to build a WeChat group. The doctor's questions were sent to the group, and we immediately checked the relevant information and provided answers.” (P1)*

“*Pharmaceutical care in the makeshift hospitals was special. Each clinical pharmacist was responsible for a dozen patients and contacted indirectly via Wechat, which was very well-done.” (P4)*

#### Subtheme 3.4-Outpatient Drug Network Delivery Service

The internet purchase and dispatch of medicines, especially prescription drugs, is strictly regulated in China. However, during the epidemic outbreak, when many patients with chronic illnesses required long-term medications and some new severe patients urgently needed medicines, hospitals around the country launched web-based consultation services. Accordingly, hospital pharmacists also started outpatient drug web-mailing services.

“*The hospital's online consultation system had embedded the cloud pharmacy so that patients could request prescriptions online and purchase medicines through the cloud pharmacy. During the epidemic, our highest number of deliveries reached over 500 a day.” (P7)*

“*Patients purchased their medicines online, and the medications were sent to their homes through courier companies, providing excellent services for the patient.” (P1)*

“*The hospital's smart pharmacy provided drug delivery services for patients during an epidemic” (P10)*

### Theme 4: Drug Management Loopholes

Our interviews revealed that managing narcotic drugs and donated medicines was one of the main problems in managing hospital drugs during an emergency.

#### Subtheme 4.1-Problems With Narcotic Drugs

Interviewees described the lack of immediate availability of narcotic drugs, the inability to collect and store paper prescriptions, and the lack of prompt collection of used empty ampoules.

“*There was no special policy for the management of narcotic drugs during the COVID-19 epidemic, and cancer pain patients still had to go to the hospital to prescribe narcotic drugs for pain relief.” (P11)*

“*Narcotic prescriptions were issued online, and paper copies were left in the ward.” (P2)*

“*Normally, we should recycle used empty ampoules of narcotic drugs to the pharmacy, but empty ampoules from isolation wards could not be recycled because they were contaminated during the COVID-19 epidemic.” (P9)*

#### Subtheme 4.2-Loopholes in the Management of Donated Medicines

During the epidemic, drugs were donated to designated hospitals and makeshift hospitals in Wuhan. However, interviewees described loopholes in managing these donated drugs.

“*Many companies showed their love by donating medicines to us, which then was still a big workload. We had to sort, store, and keep them, but they were difficult to manage, and there were no clear rules and regulations to follow.” (P1)*

“*There were inappropriate and confusing varieties of donated medicines. Lacking proper channels for donated medicines resulted in the waste of resources and the taking up human and material resources.” (P4)*

### Theme 5: Areas of Improvements of Pharmacy Services During a Pandemic

The interviewees described several inadequate pharmacy emergency response capacities. In addition, the clinical service level of pharmacists was lacking compared with that of other front-line clinical staff.

“*In the early stages of the outbreak, pharmacists were not involved in the design of drug regimen.” (P10)*

“*Some medicines could not be mailed (refrigerated drugs, narcotic drugs, and psychotropic drugs), which was also a problem for us because patients needed them. How to deal with the problem of broken drugs during transportation and how to return them? Those problems had not been solved.” (P7)*

“*Pharmacists lacked professional competence and clinical experience, and were not integrated into the treatment team.” (P4)*

“*Pharmacists might need to learn more about the treatment of diseases.” (P7)*

## Discussion

To our knowledge, this is the first multicenter national study on the delivery of clinical pharmacy services, and associated adaptations, during the COVID-19 pandemic in China. Qualitative data were obtained from 11 pharmacists from geographically diverse regions of COVID-designated hospitals. Pharmacists adapted their existing roles and implemented innovations to current work practices to counter the challenges presented by COVID-19.

The quarantines, “lockdown” measures, and social distancing caused by COVID-19 have brought considerable challenges to the supply chains of medicines worldwide. A recent global survey reported that a decrease in drug supply (69.0%) and, at the same time, a price increase (95.3%) globally involving alcohol, cannabis, prescription opioids, and sedatives (17). The shortage of medicines is more severe in underdeveloped countries such as Nigeria (18) and Rwanda (19). Therefore, governments of various countries have actively taken measures to ensure the supply of medicines during the COVID-19 pandemic (20). Among them, the US government spent billions of dollars on improving its pharmaceutical manufacturing capacity to ensure its drug supply chains (21). In the present study, many interviewees stated that their hospitals spent significant efforts in ensuring emergency medicine supply, including production, transportation, storage, and management, which were consistent with the research results of some Chinese scholars (22–25). These studies suggested that after the epidemic outbreak, Chinese hospital pharmacists quickly adjusted the supply of emergency drugs according to the pandemic change (22, 23) and established a drug distribution system in each makeshift hospital and local COVID-19 designated hospital, which basically solved the drug supply problem.

It is worth noting that the results of this study revealed that the supply of TCMPs is of significant value. Some TCMPs have been reported to benefit patients with COVID-19 (26). Recent experiment studies provide more detailed insight into the potential mechanisms underlying the therapeutic effect of TCMPs (27). The Chinese government and medical experts have also given sufficient support and attention to the clinical application of TCMPs in managing COVID-19 (28). This study showed that at the beginning of the outbreak, some drugs that the government recommended were not widely used in treating COVID-19 patients, which led to the hoarding and waste of medicines. Therefore, the emergency drug supply system should be optimized in future public health crises.

During the COVID-19 epidemic, interviewees used social media such as WeChat, video software, and official hospital websites to provide the public with timely scientific knowledge on COVID prevention and treatment. These widespread scientific messages helped dispel misconceptions and reduce public fear about COVID, consistent with the findings of other published studies (24, 25). To ensure patients' medication needs during quarantine, pharmacists provided medicines to the public through the internet. Patients had remote consultations with physicians over the hospital website. Pharmacists reviewed the prescriptions online, dispensed the medication, and arranged home deliveries. After receiving the medication, patients with medication-related inquiries can consult with pharmacists online through the social media apps (29–32). In this study, most of the respondents indicated that “internet pharmaceutical care” had the opportunity to expand during the epidemic control period and should have a broader application prospect in the future. Affected by COVID-19, pharmacists worldwide have also provided internet pharmacy services (29–32).

To control the COVID spread, pharmacists' access to the isolation ward to manage narcotic drugs was restricted. Interviewees applied several novel measures to address narcotic drugs and Class II psychotropic substances used in the isolation ward. These measures were (1) the isolation ward was temporarily approved for storing a fixed quantity of narcotic drugs, (2) narcotic drugs were changed from paper prescriptions to electronic prescriptions, and (3) video cameras were used to monitor the destruction of unused narcotics and to reconcile the amount administered. At the same time, this study uncovered several problems in managing narcotic and psychotropic drugs, such as a lack of a closed-loop for the use and retrieval of narcotic drugs in isolated wards. Our study is the first to describe Chinese pharmacists' management of narcotic drugs during the COVID-19 pandemic. A UK study investigated the impact of the COVID-19 pandemic on opioid utilization and safety (33). These studies support the need to continue surveillance of the effects of the COVID-19 pandemic on opioid utilization and safety and develop feasible emergency plans for managing narcotic drugs to meet the emergency needs in the event of a pandemic. The issue of managing donated medications was also reported in this study. Donated medicines need to be sorted, stored, and safeguarded, a significant workload for pharmacists. Excessive and inappropriate varieties of donated drugs also result in a waste of resources.

## Strengths and Limitations

To our knowledge, this is the first national study investigating pharmacists' experiences of their responses to COVID-19. The study also uncovers several future areas of improvements in pharmacy services when responding to public health crises, such as closing drug management loopholes and improving pharmacists' clinical and professional expertise in disease management. The results present insightful implications for clinical pharmacy education and training in China. Thus, the study fills in a gap in the current literature. The study has several limitations. The study findings may not be generalizable to all pharmacists and pharmacy services due to the nature of the qualitative design. Although we aimed to select pharmacists from diverse geographic locations in China, the study had a small sample size due to time and resource constraints, although data saturation was reached. The study utilized two languages. Although we have tried our best to improve the validity and minimize the risk of losing meaning, there was still the possibility of ambiguity in language conversion.

## Conclusion

This multicenter qualitative study of Chinese hospital pharmacists' experiences provided a realistic picture of how Chinese hospital pharmacists practiced during the COVID-19 pandemic. Chinese hospital pharmacists played essential roles in securing drug supply and specific pharmaceutical care activities. However, the need to improve emergency response capacity and close drug management loopholes are noted.

## Data Availability Statement

The original contributions presented in the study are included in the article/Supplementary Material, further inquiries can be directed to the corresponding author.

## Ethics Statement

The studies involving human participants were reviewed and approved by the Institutional Review Board of the First Affiliated Hospital of Zhengzhou University. The patients/participants provided their written informed consent to participate in this study.

## Author Contributions

XZ, ZY, and MW: conception, design, and manuscript writing. XZ, JL, and SD: administrative support. MW, XJ, ZY, and WZ: collection and assembly of data. MW, XJ, ZY, WZ, and XZ: data analysis and interpretation. All authors: final approval of manuscript.

## Conflict of Interest

The authors declare that the research was conducted in the absence of any commercial or financial relationships that could be construed as a potential conflict of interest.

## Publisher's Note

All claims expressed in this article are solely those of the authors and do not necessarily represent those of their affiliated organizations, or those of the publisher, the editors and the reviewers. Any product that may be evaluated in this article, or claim that may be made by its manufacturer, is not guaranteed or endorsed by the publisher.
