# Supplementary material for: Exploring the Pharmaceutical Care of Pharmacists in China During COVID-19—A National Multicenter Qualitative Study
Source: Front Public Health. 2022 Jan 27;9:797070. doi: 10.3389/fpubh.2021.797070 (PMC8829323; doi:10.3389/fpubh.2021.797070)
Supplement: Supplementary file 1 [file Table_1.DOCX]

**The checklist of the research**

**Table 1** Consolidated criteria for reporting qualitative studies (COREQ): 32-item checklist

| No Item | Guide questions/description | Answers |
| --- | --- | --- |
| **Domain 1: Research team and reflexivity** | | |
| Personal Characteristics | | |
| 1. Interviewer/facilitator | Which author/s conducted the interview or focus group? | Mingxia Wang and Zhao Yin |
| 2. Credentials | What were the researcher’s credentials? E.g. PhD, MD | MD (Zhao Yin) or MD (Mingxia Wang) |
| 3. Occupation | What was their occupation at the time of the study? | Pharmacist |
| 4. Gender | Was the researcher male or female? | Female (Mingxia Wang) and Male (Zhao Yin) |
| 5. Experience and training | What experience or training did the researcher have? | Theoretical training and experience of conducting several qualitative studies with other groups. |
| Relationship with participants | | |
| 6. Relationship established | Was a relationship established prior to study commencement? | Yes |
| 7. Participant knowledge of the interviewer | What did the participants know about the researcher? e.g. personal goals, reasons for doing the research | Reasons for doing the research |
| 8. Interviewer characteristics | What characteristics were reported about the interviewer/facilitator? e.g. Bias, assumptions, reasons and interests in the research topic | Reasons and interests in the research topic |
| **Domain 2: study design** | | |
| Theoretical framework | | |
| 9. Methodological orientation and Theory | What methodological orientation was stated to underpin the study? e.g. grounded theory, discourse analysis, ethnography, phenomenology, content analysis | Phenomenology. |
| Participant selection | | |
| 10. Sampling | How were participants selected? e.g. purposive, convenience, consecutive, snowball | Purposive and convenience. |
| 11. Method of approach | How were participants approached? e.g. face-to-face, telephone, mail, email | Face-to-face interview,telephone, |
| 12. Sample size | How many participants were in the study? | 11 |
| 13. Non-participation | How many people refused to participate or dropped out? Reasons? | No |
| Setting | | |
| 14. Setting of data collection | Where was the data collected? e.g. home, clinic, workplace | Workplace. |
| 15. Presence of non-participants | Was anyone else present besides the participants and researchers? | No |
| 16. Description of sample | What are the important characteristics of the sample? e.g. demographic data, date | Yes, demographic data. |
| Data collection | | |
| 17. Interview guide | Were questions, prompts, guides provided by the authors? Was it pilot tested? | Yes |
| 18. Repeat interviews | Were repeat interviews carried out? If yes, how many? | No |
| 19. Audio/visual | Did the research use audio or visual recording to collect the data? | Audio recording |
| 20. Field notes | Were field notes made during and/or after the interview or focus group? | Yes |
| 21. Duration | What was the duration of the interviews or focus group? | 25-70 min |
| 22. Data saturation | Was data saturation discussed? | Yes |
| 23. Transcripts returned | Were transcripts returned to participants for comment and/or correction? | Yes |
| **Domain 3: analysis and findingsz** | | |
| Data analysis | | |
| 24. Number of data coders | How many data coders coded the data? | Two |
| 25. Description of the coding tree | Did authors provide a description of the coding tree? | No |
| 26. Derivation of themes | Were themes identified in advance or derived from the data? | Derived from the data |
| 27. Software | What software, if applicable, was used to manage the data? | NVIVO 12 |
| 28. Participant checking | Did participants provide feedback on the findings? | Yes |
| Reporting | | |
| 29. Quotations presented | Were participant quotations presented to illustrate the themes / findings? Was each quotation identified? e.g. participant number | Yes |
| 30. Data and findings consistent | Was there consistency between the data presented and the findings? | Yes |
| 31. Clarity of major themes | Were major themes clearly presented in the findings? | Yes |
| 32. Clarity of minor themes | Is there a description of diverse cases or discussion of minor themes? | Yes |
