# Supplementary material for: Exploring the Pharmaceutical Care of Pharmacists in China During COVID-19—A National Multicenter Qualitative Study
Source: Front Public Health. 2022 Jan 27;9:797070. doi: 10.3389/fpubh.2021.797070 (PMC8829323; doi:10.3389/fpubh.2021.797070)
Supplement: Supplementary file 2 [file Data_Sheet_1.docx]

**Box 1. Questions used in the interview guide**

**Q1: What tasks did pharmacists undertake in fighting the COVID-19 outbreak?**

- What did you do as a pharmacist during the COVID-19 pandemic?

**Q2:** **What were the differences between your work content and style during the outbreak compared with regular times?**

- How had the coronavirus outbreak affected your work?
- As a pharmacist, what were the changes in your work style during this pandemic?

**Q3: What could hospital pharmacists do differently to improve services if time could turn back?**

- Please tell us the weakness in your work during the COVID-19 period.
- As a pharmacist, what will you do if you face a similar pandemic in the future?
